# Physiological and Molecular Changes in Cherry Red Tobacco in Response to Iron Deficiency Stress

**DOI:** 10.3389/fpls.2022.861081

**Published:** 2022-03-22

**Authors:** Fei Liu, Yihan Zhang, Xiaojun Pu, Nan Cai, Xueyi Sui, Zed Rengel, Qi Chen, Zhongbang Song

**Affiliations:** ^1^Yunnan Academy of Tobacco Agricultural Sciences, Kunming, China; ^2^Faculty of Life Science and Technology, Kunming University of Science and Technology, Kunming, China; ^3^UWA School of Agriculture and Environment, The University of Western Australia, Perth, WA, Australia; ^4^Institute for Adriatic Crops and Karst Reclamation, Split, Croatia

**Keywords:** tobacco, Cherry Red, chlorophyll, iron deficiency, transcriptome

## Abstract

The genotype CR60 is a spontaneous Cherry Red variant (containing granular red dapples on flue-cured leaves) of the Yunyan 87 (Y87) tobacco; it accumulates higher concentration of iron (Fe) in leaves than Y87, but the physiological differences between them remain largely unknown. We investigated the physiological and molecular mechanisms of CR60 in response to Fe deficiency under hydroponic conditions. Our results showed no significant phenotypic difference between Y87 and CR60 at optimal (40 μM) and high Fe (160 and 320 μM) concentrations. By contrast, CR60 exhibited higher tolerance to Fe deficiency (0 μM) than Y87, as shown by higher concentrations of chlorophyll in CR60 leaves after 21-day Fe-deficiency stress. Transcriptome profiling coupled with RT-PCR analyses found that the expression of *IRT1* and several genes associated with chlorophyll biosynthesis and photosynthesis (e.g., *PRO*, *GSA*, *FD1*, *PsbO*, and *PC*) was higher in CR60 than Y87. These results indicated that CR60 maintains sufficient Fe uptake, chlorophyll biosynthesis and photosynthetic rate when subjected to Fe starvation.

## Introduction

Iron (Fe) is an essential plant micronutrient. The main valence forms are Fe (II) and Fe (III) (Ilbert and Bonnefoy, 2013). Although the total iron concentration in soils is high, it exists mostly in the oxidized Fe (III) form in aerated soils; this form is not directly plant-available (Guerinot and Yi, 1994; Briat et al., 2015). Plants have evolved two different molecular strategies to obtain sufficient Fe from the rhizosphere to adapt to the low-Fe environments. Dicots and non-graminaceous monocots have evolved different Fe uptake mechanisms (strategy I) (Romheld and Marschner, 1986; Hell and Stephan, 2003; Kobayashi and Nishizawa, 2012) compared with graminaceous plants (strategy II) (Romheld and Marschner, 1986; Connorton et al., 2017). Strategy I plants (such as tobacco) secrete phenolic compounds *via* phenolics efflux transporters and pump H^+^ out into the rhizosphere *via* the plasma membrane H^+^-ATPase, thus increasing iron solubility by chelation and contributing to Fe^3+^ reduction *via* acidification (Kim and Guerinot, 2007). Upon transfer of the dissolved Fe^3+^ from the rhizosphere to the root surface, ferric chelate reductase (FRO) reduces it to Fe^2+^ (Robinson et al., 1999), and then Fe^2+^ is absorbed into root cells by high-affinity iron-regulated transporters (IRTs) (Eide et al., 1996; Vert et al., 2002; Brumbarova et al., 2015).

Cellular iron combines with various organic substances as a structural/functional component, such as in cytochromes, catalase, peroxidases, heme, ferredoxin, and iron-sulfur clusters (Balk and Schaedler, 2014; Vigani and Murgia, 2018); these compounds are essential in chlorophyll synthesis, oxygen transport, redox reactions, nitrogen assimilation, photosynthesis, and electron transport in plants (Balk and Schaedler, 2014). For example, heme, cytochrome, iron-sulfur clusters, and ferredoxin are key components of the photosynthesis system and contribute to the photosynthetic and respiratory electron transport chains (Sousa et al., 2018; Kroh and Pilon, 2020). Therefore, iron deficiency not only leads to the inhibition of chlorophyll synthesis and diminished photosynthetic efficiency (Nelson and Ben-Shem, 2004; Msilini et al., 2011), but also results in the interruption of the respiratory electron transport in plant mitochondria and tricarboxylic acid cycle metabolism, hampering plant growth (Fernie et al., 2004).

Flue-cured leaves from common commercial tobacco [e.g., Yunyan87 (Y87)] usually display yellow appearance. However, a type of spontaneous variant that originated from flue-cured varieties has a reddish hue in flue-cured leaves and has historically been referred to as Cherry Red tobacco (Wada, 1956; Hall et al., 1965). It is reported that Cherry red leaves contain high levels of nornicotine as the principal alkaloid, whereas nicotine is the principal alkaloid in common tobacco with flue-cured yellow leaves (Wada, 1956; Hall et al., 1965). Compared with the common tobacco, high concentrations of nornicotine in the Cherry Red tobacco leaves are associated with a strong expression of nicotine *N*-demethylase gene *CYP82E4*, which plays a key role in nicotine to nornicotine conversion (Siminszky et al., 2005). Nornicotine was suggested to be a substrate reacting with *o*-quinones (produced by oxidation of chlorogenic acid and caffeic acid by polyphenol oxidase) to generate red color (570 nm wavelength) in Cherry Red tobacco (Weeks et al., 1993).

During several years of field observations, we identified a spontaneous Cherry Red variant (CR60) that originated from flue-cured cultivar Yunyan87 (Y87). In CR60, the *CYP82E4* expression was activated by an unknown mechanism, resulting in significantly increased accumulation of nornicotine in leaves (Song et al., 2021), similarly to the previous report (Siminszky et al., 2005). However, apart from *CYP82E4* expression and correspondingly high levels of nornicotine, the growth characteristics and physiological/molecular properties of CR60 are largely unknown.

In this study, we found that CR60 accumulates higher concentrations of iron in comparison with Y87 under field conditions, implying the difference in iron uptake and transport between the two genotypes. Therefore, we further analyzed the physiological and molecular changes in CR60 and Y87 exposed to different concentrations of iron under hydroponic conditions. The results showed that CR60 exhibited higher iron deficiency tolerance than Y87, which might be attributed to higher expression of several genes associated with iron uptake, chlorophyll biosynthesis and electron transport chain in the photosynthetic system of CR60.

## Materials and Methods

### Plant Growth and Treatment

Seeds of CR60 and Y87 were germinated in a mixture (vermiculite:perlite:peat soil = 1:1:1, v:v:v) at 22°C under cool white light (100 μmol m^–2^ s^–1^) with the 12-h-light/12-h-dark cycle. Ten days after germination, the seedlings were transferred to pots containing 1 L of 1/2-strength Hoagland nutrient solution (pH 6.0) with 40 μM Fe (III)-EDTA. After 3 days of recovery growth, the seedlings were transferred to fresh 1/2-strength Hoagland nutrient solution (pH 6.0) with different treatment concentrations of Fe (III)-EDTA (0, 40, 160, and 320 μM). The nutrient solutions were replaced every 2 days.

### Measurement of Chlorophyll Contents

Chlorophyll was extracted from the first fully extended mature leaves (0.1 g) using dimethyl sulfoxide (DMSO, 1.5 mL) at 65°C under dark. Then, 8 mL acetone was added and mixed thoroughly. After centrifugation at 12,000 *g* at 4°C for 20 min, the supernatant was used for measurement of chlorophylls *a* and *b* using a spectrophotometer at 663 and 646 nm, respectively. The concentrations of chlorophylls *a* and *b* and total chlorophyll were calculated as described elsewhere (Hiscox and Israelstam, 1980; Lichtenthaler and Buschmann, 2001).

### Total RNA Isolation, Library Construction and RNA Sequencing

Total RNA was isolated from the first fully expanded apical leaves of tobacco using the Trizol Reagent (Invitrogen, United States). A total amount of 1 μg RNA per sample was used as input material for the RNA-Seq sample preparations. Sequencing libraries were generated using a NEBNext^®^ Ultra™ RNA Library Prep Kit for Illumina (NEB, United States) following the manufacturer’s instructions. The mRNA was purified from total RNA using poly-T oligo-attached magnetic beads. Fragmentation was carried out using divalent cations under elevated temperature in the NEBNext First Strand Synthesis Reaction Buffer (5X). The first strand of cDNA was synthesized using the fragmented mRNA as a template and random oligonucleotides as primers in a M-MuLV reverse transcriptase system, followed by the degradation of the RNA strand by RNase H. The second strand of cDNA was synthesized using dNTPs in a DNA polymerase I system. The purified double-stranded cDNA was end repaired and A-tailed, sequencing adaptors were ligated, and approximately 200 bp of cDNA was screened with AMPure XP beads (Beckman Coulter, Beverly, MA, United States). After PCR amplification, the PCR products were purified with AMPure XP beads, and the library was obtained. Library quality was assessed on an Agilent Bioanalyzer 2,100 system. Twelve libraries named Y87_0 and CR60_0 (0 μM Fe) and Y87_40 and CR60_40 (40 μM Fe), each in three biological replicates (A, B, and C), were constructed. The libraries were then sequenced on an Illumina Novaseq platform, and 150 bp paired-end reads were generated. The experiments were conducted three times. The RNA sequence dataset is available in the repository of NCBI Sequence Read Archive (SRA) with the GeneBank accession No.: PRJNA807089 (https://www.ncbi.nlm.nih.gov/sra/?term=PRJNA807089).

### Bioinformatic Analysis

High-quality clean reads were obtained by removing the adaptor sequences, unclear “N” nucleotides and low-quality sequences from raw reads. HISAT2 (v2.0.5) software was used to establish the index of the tobacco reference genome and align the clean reads to the tobacco reference genome (Mortazavi et al., 2008).

The featureCounts (v1.5.0-p3) was used to count the reads numbers mapped to each gene (Liao et al., 2014). The gene expression levels were represented by the expected number of Fragments Per Kilobase of transcript sequence per Million base pairs sequenced (FPKM), which was calculated on the length of the gene and the reads count mapped to this gene. Differential expression analyses were performed using the DESeq2 R package (v1.16.1). Genes with an adjusted *P*-value < 0.05 and | log2(FoldChange)| > 0 were considered to be differentially expressed. The Padj values were adjusted using the Benjamini and Hochberg method for controlling the false discovery rate (FDR). Gene Ontology (GO) and KEGG enrichment analysis of differentially expressed genes was implemented by the clusterProfiler and ggplot2 R packages (v3.4.4). The GO and KEGG terms with corrected *P*-value < 0.05 were considered significantly enriched in differentially expressed genes.

### Reverse Transcription-Polymerase Chain Reaction Analysis

Total RNA was extracted using RNAiso Plus (Takara, Da Lian, China) according to the manufacturer’s instructions. Reverse transcription was performed using a PrimeScript RT reagent kit with gDNA eraser (Takara) following the manufacturer’s instructions. Quantitative PCR reactions were performed in 20 μL reaction volumes using a SYBR Premix EX Taq II Kit (Takara) according to the manufacturer’s instructions. The primers used in this study are listed in Supplementary Table 1.

## Results

### CR60 Leaves Accumulate High Concentrations of Iron

**FIGURE 1:**
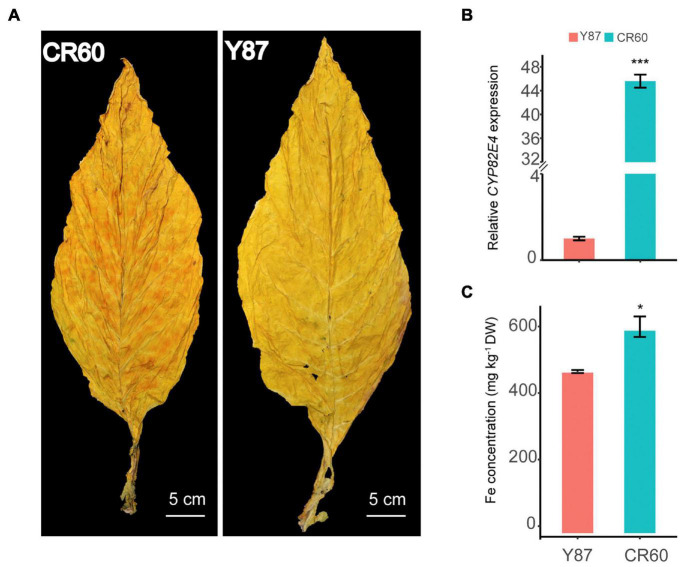
Differential appearance of flue-cured leaves **(A)**, expression of *CYP82E4* gene **(B)** and Fe concentrations **(C)** in leaves of CR60 and Y87. The plants were cultivated in an experimental field located at the Yanhe Research Farm of Yunnan Academy of Tobacco Agricultural Sciences, Yunan Province, China (N24.14, E102.30, altitude 1,680 m). For panel **(A)** the matured leaves were harvested and flue-cured following local agricultural practices. For panel **(B)**, leaves at stalk position #10 (from top to bottom) of 2-month-old plants were harvested for RNA isolation. For panel **(C)** the 5th to 10th (from top to bottom) leaves were flue-cured and mixed for ICP-MS analysis. Three biological replicates were analyzed. Values are means ± SD (*n* = 3). Asterisks indicate significant differences between the CR60 and Y87 plants (*P < 0.05 and ***p < 0.001) as determined by the Student’s *t*-test.

Granular red dapples were observed on leaves of CR60, but not in Y87 after curing (Figure 1A). Additionally, the expression of *CYP82E4*, a key gene involved in the conversion of nicotine to nornicotine (Siminszky et al., 2005), was significantly higher in CR60 than Y87 (Figure 1B). In field conditions, CR60 and Y87 did not show obvious differences in plant height, root length, leaf number, and biomass (data not shown); however, higher concentrations of iron were recorded in CR60 leaves compared with Y87 (Figure 1C).

### CR60 Showed Higher Iron Deficiency Tolerance Than Y87

**FIGURE 2:**
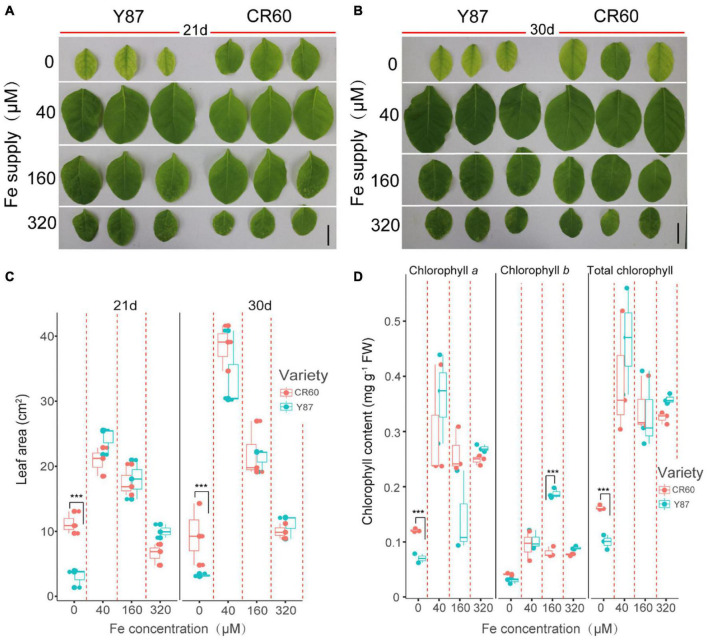
CR60 had higher iron deficiency tolerance than Y87. Images **(A,B)**, leaf area **(C)** and chlorophyll concentrations **(D)** of the first fully expanded apical leaves of CR60 and Y87 after growing at different nutrient solution concentrations of iron (0, 40, 160, and 320 μM) for 21 and 30 days. The experiment was conducted at least three times and showed similar results. For panels **(C,D)** data were collected from plants grown at differential iron supply for 21 days and were expressed as means ± SD (*n* = 3). Asterisks indicate statistically significant differences between CR60 and Y87 (***p < 0.001) as determined by the Student’s *t*-test.

We then examined the physiological responses of CR60 and Y87 to differential supply of iron under hydroponic conditions. High solution concentrations of iron (160 and 320 μM) inhibited leaf growth of CR60 and Y87, but there were no significant differences between these two genotypes (Figures 2A–C). By contrast, the first fully expanded apical leaves of Y87 exhibited obvious interveinal chlorosis after stressed by iron deficiency for 21 and 30 days, whereas CR60 plants developed larger leaf area and greener first expanded leaves than Y87 under the same stress (Figures 2A–C). In accordance with these observations, concentrations of chlorophyll *a* and the total chlorophyll in the first fully expanded apical leaves of CR60 were, respectively, 42 and 44% higher than those in Y87 after the plants were stressed by iron deficiency for 21 days (Figure 2D).

### Differentially Expressed Genes Analysis of CR60 and Y87 Between Optimal and Deficient Iron Supply

**FIGURE 3:**
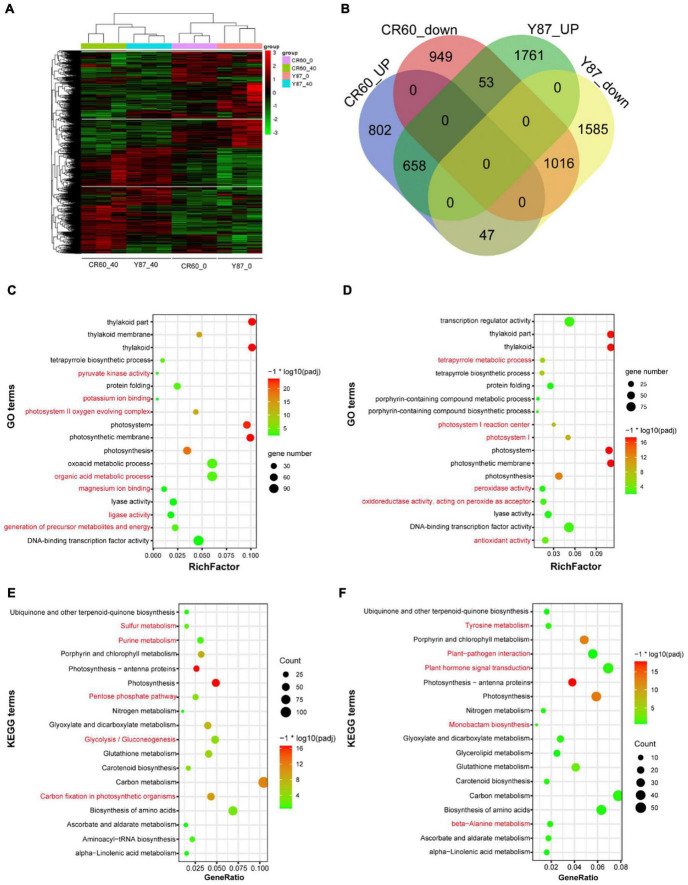
Transcriptome profiling of CR60 and Y87 in response to iron deficiency. **(A)** Hierarchical cluster analysis of differentially expressed genes (DEGs) of CR60 and Y87 under optimal and iron deficiency conditions (*n* = 3). **(B)** Venn diagram showing upregulated and downregulated DEGs in CR60 and Y87 stressed by iron deficiency for 21 days compared with plants grown under optimal conditions (*n* = 3). The GO (Gene Ontology) enrichments of DGEs in Y87 **(C)** and CR60 **(D)**. The KEGG (Kyoto Encyclopedia of Genes and Genomes) enrichments of DGEs in Y87 **(E)** and CR60 **(F)**. Plants were grown hydroponically in 1/2 Hoagland nutrient solutions containing 0 or 40 μM Fe (III)-EDTA for 21 days. The first fully expanded apical leaves were harvested for RNA isolation and RNAseq analysis (*n* = 3).

Given the different changes in the first fully expanded leaves between CR60 and Y87 under iron deficiency conditions, we performed RNA sequencing (RNA-seq) to compare the transcriptome changes in the two genotypes under optimal and iron deficiency conditions. Iron deficiency treatment triggered extensive transcriptome changes in both CR60 and Y87 (Figure 3A). Compared with plants grown under optimal conditions [40 μM Fe (III)-EDTA], iron deficiency [0 μM Fe (III)-EDTA] upregulated 1,507 (43%) and 2,472 (48%) differentially expressed genes (DEGs) in CR60 and Y87, respectively, whereas 2018 (57%) and 2648 (52%) DGEs were downregulated, respectively (Figure 3B). The DGEs showed similar patterns in both CR60 and Y87, and were highly related to photosynthesis, chlorophyll metabolism, carbon metabolism, plant hormone signaling, and peroxidase and oxidoreductase activity (Figures 3C–F and Supplementary Tables 2–5).

### Iron Starvation Regulated Gene Expression Differently in CR60 and Y87

**FIGURE 4:**
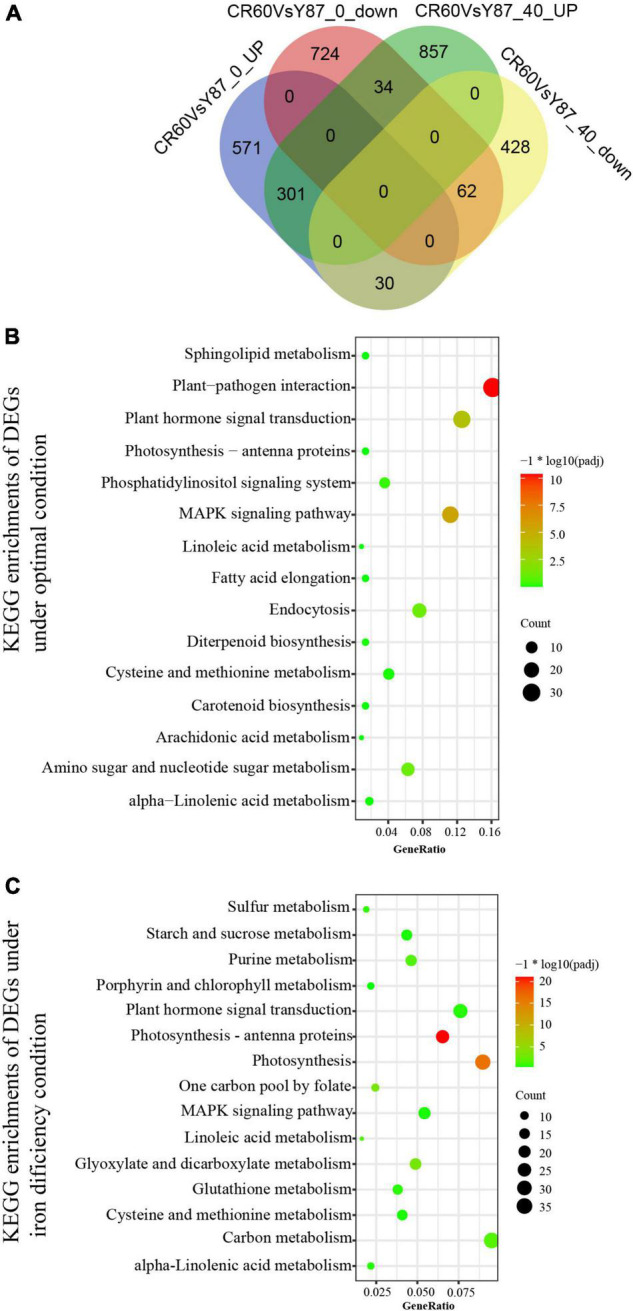
Comparison of iron starvation-regulated transcriptome between CR60 and Y87. **(A)** Venn diagram showing DEGs between CR60 and Y87 under optimal and iron deficiency conditions. KEGG enrichments of DEGs under optimal conditions **(B)** or iron deficiency **(C)**.

Under optimal growth conditions [40 μM Fe (III)-EDTA], 1,712 differentially expressed genes (DEGs) were identified in CR60 in comparison with Y87, out of which 1,192 (69.6%) were upregulated and 520 (30.4%) were downregulated (Figure 4A). These DEGs were involved mostly in plant pathogen interaction, MAPK signaling, endocytosis, sugar metabolism, and plant hormone signaling (Figure 4B). Under Fe deficiency conditions, 902 (52.4%) upregulated DEGs and 820 (47.6%) downregulated DEGs were found in CR60 in comparison with Y87 (Figure 4A). The DEGs upregulated by iron deficiency were enriched predominantly in photosynthesis (numbers of KEGG terms increased from three to 57), photosynthetic antenna proteins, carbon metabolism, starch and sucrose metabolism, glyoxylate and dicarboxylate metabolism, and purine metabolism (Figure 4C).

### Expression Profiles of Genes Associated With Iron Uptake and Homeostasis

**FIGURE 5:**
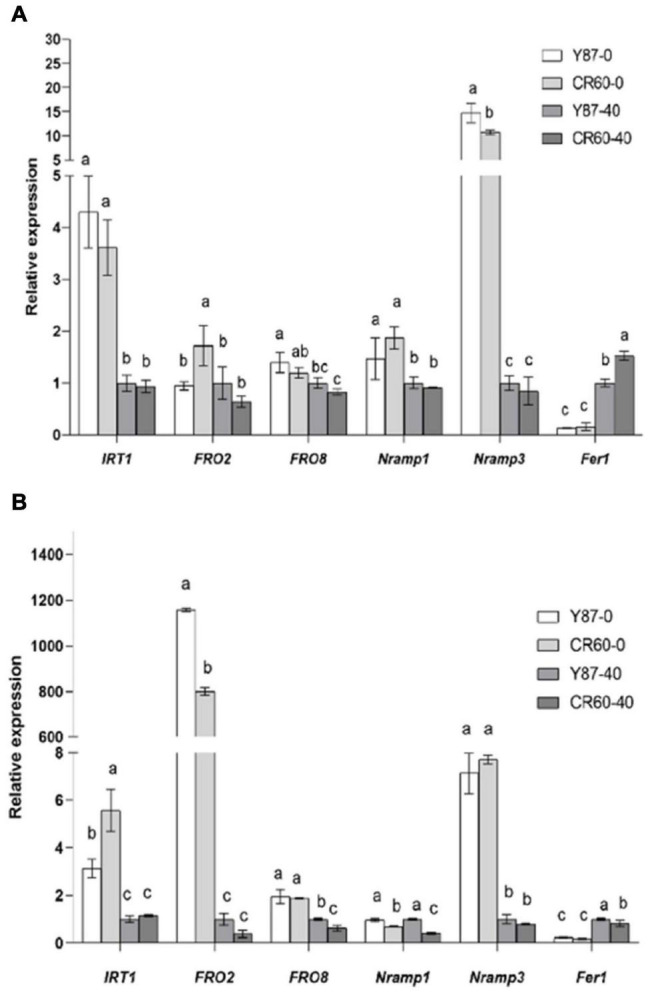
Relative expression of genes associated with iron uptake and homeostasis in leaves **(A)** and roots **(B)** of Y87 and CR60. The plants were grown in 1/2 Hoagland nutrient solutions containing 0 or 40 μM Fe (III)-EDTA for 21 days. The first fully expanded apical leaves or whole roots were harvested for RNA isolation and RT-PCR analysis. The data are expressed as means ± SD (*n* = 6). Different letters indicate significant differences at *P* < 0.05 (Student’s *t*-test).

The expression of *IRT1* (a high-affinity Fe^2+^-transporter) and *NRAMP3* (natural resistance-associated macrophage protein 3) was induced, but *Fer1* (ferritin 1, coding for Fe storage protein) was downregulated by Fe deficiency in leaves and roots of Y87 and CR60 (Figures 5A,B). The upregulation of *FRO2* (a ferric-chelate reductase) and *FRO8* in roots or *NRAMP1* in leaves of Y87 and CR60 was observed. Although the expression of *IRT1* in leaves did not show significant difference between Y87 and CR60 under normal growth conditions, its expression was significantly higher in CR60 roots than Y87 roots under Fe deficiency conditions (Figures 5A,B). Additionally, *Fer1* showed higher expression in CR60 than Y87 leaves at optimal Fe concentration (Figures 5A,B).

### Expression Profiles of Genes Associated With Chlorophyll Biosynthesis and Photosynthesis

**FIGURE 6:**
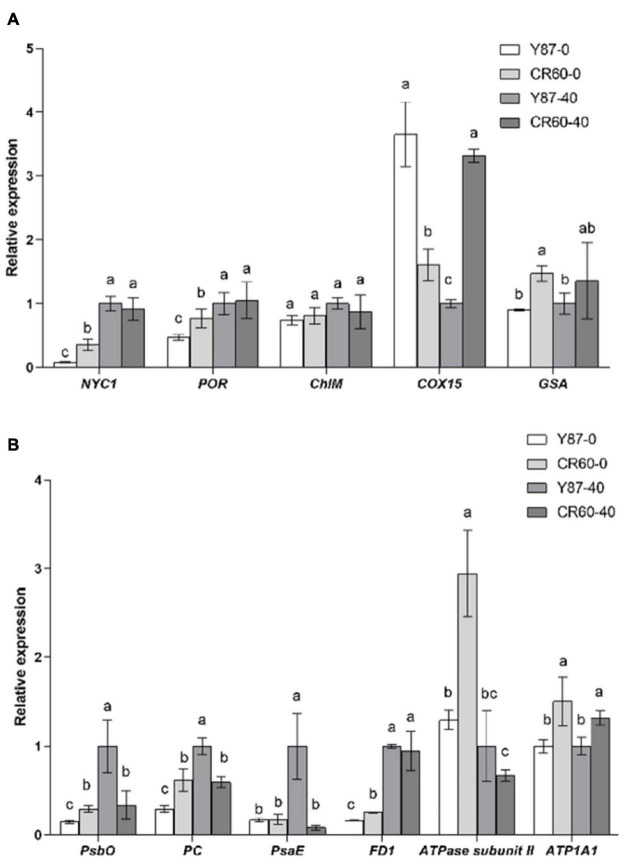
Expression of genes associated with chlorophyll synthesis **(A)** and photosynthesis **(B)** in the first fully expanded apical leaves of Y87 and CR60. The plants were grown in 1/2 Hoagland nutrient solutions containing 0 or 40 μM Fe (III)-EDTA for 21 days. The data are expressed as means ± SD (*n* = 3). Different letters indicate significant differences at *P* < 0.05 (Student’s *t*-test).

Several genes encoding key enzymes involved in chlorophyll metabolism, including *NYC1* (chlorophyll *b* reductase) and *POR* (protochlorophyllide oxidoreductase) were reduced slightly by iron deficiency, and the relative expression of these two genes was higher in CR60 than Y87 (Figure 6A). The expression of *COX15* (cytochrome c oxidase assembly protein) was higher in CR60 than Y87 under optimal growth conditions; by contrast, *COX15* was decreased by Fe deficiency in CR60, but increased in Y87. Additionally, the expression of *GSA* (glutamate 1-semialdehyde aminotransferase) in leaves was higher in CR60 than Y87 under both optimal and Fe deficiency conditions.

The expression of several genes associated with photosystems I and II and the electron transport chain was also analyzed (Figure 6B). The expression of *PsbO* (encoding the photosystem II manganese-stabilizing protein), *PC* (encoding plastocyanin), *PsaE* (encoding photosystem I reaction center subunit IV), and *FD1* (encoding ferredoxin 1) was decreased by Fe deficiency in Y87 leaves, whereas the expression of these genes (*PsbO*, *PC*, and *PsaE*) was unaffected in CR60 under Fe deficiency. The expression of *ATPase subunit II* was significantly increased by Fe deficiency in CR60. Additionally, the expression of *PsbO*, *PC*, *FD1*, *ATPase subunit II*, and *ATPase subunit alpha* (*ATP1A1*) was higher in CR60 than Y87 under Fe deficiency (Figure 6B).

## Discussion

As a micronutrient, iron plays an irreplaceable role in regulating plant growth and metabolism (Kobayashi and Nishizawa, 2012). Iron is involved in chlorophyll synthesis and serves as a cofactor in numerous enzymes (Balk and Schaedler, 2014; Kroh and Pilon, 2020). It has long been known that the photosynthetic capacity of higher plants is affected by iron deficiency. The primary response associated with the insufficient availability of iron is the loss of chlorophyll and interveinal chlorosis of young leaves (Miller et al., 2008). In this work, we found that the first fully expanded apical leaves of Y87 exhibited an obvious interveinal chlorosis after 21 days of iron deficiency treatments; by contrast, CR60 remained green and contained high concentrations of chlorophyll, indicating that CR60 is more tolerant to iron deficiency stress than Y87 (Figure 2). A comparative transcriptome analysis between CR60 and Y87 further showed that the differences in chlorophyll synthesis and photosynthesis might be responsible for their phenotypic differences (Figures 3C–F, 4B,C).

Iron uptake and translocation are tightly regulated by its transport- and homeostasis-related genes in plants. In strategy I plants (e.g., tobacco), ferric-chelate reductase (FCR) catalyzes the reduction of Fe^3+^ to Fe^2+^, which is then assimilated into root cells *via* the high-affinity metal transporter IRT1 (iron-regulated transporter 1) (Eide et al., 1996; Robinson et al., 1999; Vert et al., 2002; Santi and Schmidt, 2009). NRAMP3 is a vacuolar metal transporter involved in homeostasis and transport of divalent metals (e.g., Fe^2+^, Mn^2+^ and Cd^2+^) (Thomine et al., 2003). Additionally, ferritin localized in plastids of plant leaves is an iron storage protein, and the expression of *ferritin 1* (*Fer1*) is induced by iron application in arabidopsis (Ravet et al., 2009). In this study, we found that the expression of *FRO2* and *FRO8*, two genes encoding ferric-chelate reductase, were induced in roots of both genotypes by Fe deficiency (Figure 5B). Additionally, *IRT1* and *NRAMP3* were also upregulated by Fe starvation in both leaves and roots of the two genotypes, whereas the expression of *IRT1* was higher in roots of CR60 than Y87 (Figure 5B). Iron deficit reduced the expression of *Fer1* in leaves of both CR60 and Y87, but no significant difference was found between the two genotypes (Figure 5A). These results indicate CR60 and Y87 share similar responses regarding Fe^3+^ reduction and iron storage under iron deficiency. The higher leaf accumulation of Fe in CR60 than Y87 (Figure 1B) might be attributed to higher expression of *IRT1* in roots of CR60 than Y87 during Fe starvation (Figure 5B), which contributed to greater tolerance to iron deficiency stress in CR60 compared with Y87. In tomato plants, iron affects the expression of *SlIRT1 via* modulation of the DNA methylation of its promoter (Chen et al., 2022). Therefore, investigating whether epigenetic modulation is also involved in iron-mediated changes in the expression of these genes in tobacco is warranted.

Iron is required not only for chlorophyll biosynthesis and the maintenance of chloroplast structure and function, but also serves as an important component of cytochromes in the electron transport chain. Approximately 80% of iron is located in photosynthesizing cells and is directly required for the structural and functional integrity of thylakoid membrane and the biosynthesis of chlorophyll (Li et al., 2021). Visible chlorosis (a decrease in chlorophyll concentration) was found in Y87 leaves stressed by Fe deficiency for 21 days (Figures 2A,D), indicating that Fe starvation affected the biosynthesis of chlorophyll. In contrast, CR60 developed larger leaf area, remained greener and contained higher chlorophyll concentrations than Y87 after 21 days of Fe deficiency (Figures 2B–D). Congruent with these observations, our transcriptome and RT-PCR results showed that a large number of genes associated with photosynthesis, PS-I/II and chlorophyll metabolism were regulated by Fe deficiency (Figures 3, 4, 6 and Supplementary Tables 2–5). More specifically, the expression of *NYC1*, *PRO*, and *GSA*, key genes involved in the biosynthesis of chlorophyll and heme, was significantly higher in CR60 than Y87 under iron deficiency stress (Figure 6A), which was in accordance with higher chlorophyll concentrations measured in CR60 than Y87 (Figure 2D). Although iron starvation decreased the expression of *PsbO*, *PC*, and *FD1* (all associated with the electron transfer chain in the photosynthetic system), the relative expression of these genes was significantly higher in CR60 than Y87 (Figure 6B). Additionally, the *ATPase subunit II* expression was induced by iron deficiency and was higher in CR60 than Y87. These results suggested that CR60 maintained greater chlorophyll biosynthesis and had higher photosynthetic rate under Fe starvation than Y87.

## Conclusion

The CR60 showed higher tolerance to iron deficiency than Y87, as shown by significant differences in leaf growth and chlorophyll concentrations after 21 days of iron deficiency. The expression of *IRT1* and several genes associated with chlorophyll biosynthesis (e.g., *NYC1*, *PRO*, and *GSA*) and the electron transport chain in photosynthesis (e.g., *PsbO*, *PC*, *FD1*, *ATPase subunit II*, and *ATP1A1*) was higher in CR60 than Y87, which could at least partially explain greater tolerance to Fe starvation in CR60 than Y87. Furthermore, our preliminary data showed that the concentrations of iron in CR60 leaves was correlated positively with the development of granular red dapples, indicating iron might be also related to the formation of Cherry Red leaves. Therefore, whether and how iron regulates the formation of Cherry Red tobacco are questions worth investigating in the future studies.

## Data Availability Statement

The data presented in the study are deposited in the National Center for Biotechnology Information (NCBI) Sequence Read Archive (SRA), accession number PRJNA807089.

## Author Contributions

ZS conceived this study, analyzed the data, and wrote and revised the manuscript. FL and YZ performed the experiments, analyzed the data, and wrote the manuscript. XP, NC, and XS analyzed the data and revised the manuscript. ZR and QC analyzed the data, interpreted the results, and revised the manuscript. All authors contributed to the article and approved the submitted version.

## Conflict of Interest

The authors declare that the research was conducted in the absence of any commercial or financial relationships that could be construed as a potential conflict of interest.

## Publisher’s Note

All claims expressed in this article are solely those of the authors and do not necessarily represent those of their affiliated organizations, or those of the publisher, the editors and the reviewers. Any product that may be evaluated in this article, or claim that may be made by its manufacturer, is not guaranteed or endorsed by the publisher.

## Supplementary Material

Supplementary Table 1 Primers used in this study.

Supplementary Table 2 List of GO terms of CR60 under iron deficiency conditions.

Supplementary Table 3 List of GO terms of Y87 under iron deficiency conditions.

Supplementary Table 4 List of KEGG terms of CR60 under iron deficiency conditions.

Supplementary Table 5 List of KEGG terms of Y87 under iron deficiency conditions.
