# Supplementary material for: Physiological and Molecular Changes in Cherry Red Tobacco in Response to Iron Deficiency Stress
Source: Front Plant Sci. 2022 Mar 22;13:861081. doi: 10.3389/fpls.2022.861081 (PMC8980409; doi:10.3389/fpls.2022.861081)
Supplement: Supplementary Table 1 — Primers used in this study. [file Table_1.docx]

Supplementary Table S1 Primer sequence for fluorescence quantitative PCR

| Primers | Sequence (5’-3’) |
| --- | --- |
| CYP82E4-F | AGGGACAAGATTATTCGCAAACG |
| CYP82E4-R | ACCACGAAAGTCAATATCAATAGCA |
| FRO2-F | AGCAATCAACAACAGAAGACAAGA |
| FRO2-R | ATAGCACCAAGCCAAAGCCA |
| FRO8-F | GTGTCTTCGTTTGTGGGCCT |
| FRO8-R | AGCTGAAGGATGGCTTTTGTTC |
| Nramp1-F | TACCTCTCTCAAGGATAGCCATTT |
| Nramp1-R | CTTGTAAACCTTCTGCCCTGT |
| Nramp3-F | ACCGATTACTCGATTCCAACG |
| Nramp3-R | AAAGGAGGAGCTTCGGTGAA |
| Fer1-f | ATCATGCGATGTTTGCGTATT |
| fer1-r | GGTGGCGCACAGATAGACA |
| IRT1-F | TGTCACTCCTGAAAATGGTG |
| IRT1-R | CTTTAGAGCTTAGGGATCCG |
| NYC1-F | GTGCATGGCTTTTACCGCTG |
| NYC1-R | TGACACAATCATGCCATAGCG |
| COX15-F | TCACAAGGGTTTGCATAGAACA |
| COX15-R | CTGAAGACCCAAGCAGCAGA |
| POR-F | GTGGTGGTCCAGCAAGTCTAA |
| POR-R | TGGCAAATACGTGATGGATCATA |
| ChIM-F | TGGCCATGTTTCTTTGTGCTG |
| ChIM-R | AAATTTTTCCAAGTCAGAAGCAGA |
| GSA-F | TTTTCTCTCCATCTCCCCGC |
| GSA-R | GAGAGCCCTTGGCTTTGCTA |
| FD1F | GGCCAGTACCATGGTTAGCA |
| FD1R | CTCAACAGCTCCCTCTGGTG |
| ATPase subunitⅡ-F | AGAGGGAGGAAGATATTAGCCC |
| ATPase subunitⅡ-R | GAGGACAGTGAGCTTGAGGG |
| Atp1a1-F | TTCAAGTAGGCGACGGCATT |
| Atp1a1-R | TTCGACTAATTCACCCGCCA |
| PsbO-F | TCAAAGTGGAGCAAATGCTGA |
| PsbO-R | ATTGTTGGGCATTGGTTGGC |
| PsaE-F | CGAGGCTCGTTGTGAGAGTT |
| PsaE-R | ACAACTGGGTAGCGTGTGTT |
| PC-F | GTAGCGATGATGGCGGTCTT |
| PC-R | GTCTCCCCTGGTCCATTGAG |
| Actin-F | CTGAGGTCCTTTTCCAACCA |
| Actin-R | TACCCGGGAACATGGTAGAG |
